# Quantum Confinement Effect in a Heteromorphic PbS/SnS_2_ Superlattice Grown by Atomic Layer Deposition

**DOI:** 10.1021/acsnano.6c02718

**Published:** 2026-06-11

**Authors:** Dong-Ho Shin, Mohammadreza Daqiqshirazi, Amin Bahrami, Sebastian Lehmann, Daniel Wolf, Axel Lubk, Angelika Wrzesińska-Lashkova, Yana Vaynzof, Golam Haider, Thomas Brumme, Kornelius Nielsch

**Affiliations:** † 28394 Leibniz Institute for Solid State and Materials Research, Dresden 01069, Germany; ‡ Institute of Applied Physics, Technische Universität Dresden, Dresden 01062, Germany; § Institute of Material Research, Technische Universität Dresden, Dresden 01062, Germany; ∥ Chair for Theoretical Chemistry, 9169 Technische Universität Dresden, Dresden 01069, Germany; ⊥ Chair for Emerging Electronic Technologies, Technische Universität Dresden, Dresden 01187, Germany; # Center for Advanced Systems Understanding, CASUS, HZDR, Görlitz 02826, Germany

**Keywords:** Atomic layer deposition, Superlattice, Heteromorphic, Quantum confinement, Bandgap, PbS

## Abstract

Quantum confinement in artificial superlattices enables the engineering of electronic and optical properties that exceed bulk limitations. Despite the unrivaled precision in thickness control and scalability of atomic layer deposition (ALD), the experimental demonstration of confinement effects in the superlattice geometry has remained elusive, especially for nonoxide systems. In this study, we report the experimental demonstration of distinct quantum confinement in the chalcogenide-based heteromorphic superlattices via an ALD supercycle approach. Polycrystalline PbS and amorphous SnS_2_ are alternately deposited with subnanometer thickness control, resulting in the formation of a well-defined heteromorphic superlattice structure with sharp, strain-relieved, and defect-passivated interfaces. Raman spectroscopy also revealed the activation of low-frequency vibrational modes, indicating strong interlayer coupling of strain-free layers within the superlattice. A systematic reduction in the PbS sublayer thickness below its excitonic Bohr radius enables a substantial and controllable widening of the bandgap, from 1.74 eV for PbS (14 nm)/SnS_2_ (5 nm) to 2.51 eV for PbS (3 nm)/SnS_2_ (5 nm), compared to 1.54 eV for individually grown PbS films. This marked bandgap modulation unambiguously demonstrates the strong quantum confinement of charge carriers within the strain-relaxed PbS layers. Density functional theory (DFT) calculations confirm the experimental observations, revealing the emergence of both lateral and vertical quantum confinement and elucidating the role of the superlattice architecture in shaping the electronic structure. Together, these results establish ALD as an effective platform for quantum superlattice engineering, enabling precise control of confinement effects in complex chalcogenide heterostructures and their integration into next-generation optoelectronics and quantum devices.

## Graphical abstract

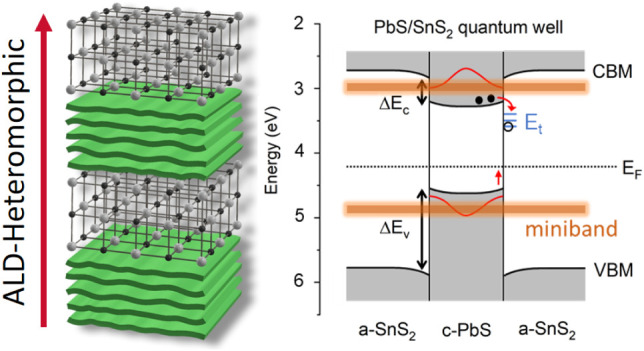

## 1 Introduction

Superlattice structuring has attracted considerable attention from researchers since it was first proposed by Esaki and Tsu in the 1970s.^1^ The ability to precisely control quantum confinement is among the most effective methods for engineering electronic and optical functionalities beyond bulk limits and has yielded significant advancements in fields such as optoelectronics,^2^ catalysis,^3^ phase-change memory,^4^ and thermoelectric technology.^5^ On the basis of electronic band alignment, superlattices are generally classified into three types. Type-I (straddling alignment) superlattices confine both electrons and holes within the same material layer. Type-II (staggered alignment) superlattices separate electrons and holes into different layers, enabling spatial charge separation. Type-III (broken gap) superlattices exhibit a band overlap where the valence band maximum of one material lies above the conduction band minimum of the other. A type-I superlattice is a periodically alternating semiconductor heterostructure that confines both electrons and holes within one sublayer. This spatial confinement results in the formation of discrete energy levels, enabling the observation of quantum size effects. These confinement-driven phenomena highlight a wide range of modern nanoscale electronic device technologies. Despite decades of progress, achieving precise, scalable, and industry-compatible control of quantum confinement in artificial superlattices remains challenging in material science.

Conventional approaches to structure superlattices, e.g., molecular beam epitaxy (MBE),^6–9^ sputtering,^10,11^ metal–organic chemical vapor deposition (MOCVD),^12,13^ and chemical vapor deposition (CVD),^14,15^ have been reported focusing on epitaxial superlattice structure. However, this approach restricts the range of material combinations due to stringent lattice matching requirements <∼ 5% and strain accumulation during growth.^16,17^ To overcome these limitations, Tsu et al. proposed a superlattice concept in which materials with different morphologies could be periodically integrated to broaden the range of accessible material systems.^18^ In this proposal, polycrystalline (*pc*) semiconductors were to be passivated with amorphous (*a*) oxides, such as pc-Si/a-SiO_2_. The amorphous oxide layer was proposed to passivate the high defect density at grain boundaries, enabling the emergence of quantum effects. They also suggested that III–V compound semiconductors combined with amorphous chalcogenides could benefit from passivating layers. Despite its conceptual significance, the practical realization of such structures remained challenging for several decades. Only recently, Lee et al. demonstrated a superlattice consisting of *pc*-In_2_O_3_/*a*-MoO_3_ layers grown by pulsed laser deposition (PLD), integrating layers with different morphologies.^19^ They introduced the term *heteromorphic superlattice* to describe such structures, which was subsequently employed by Shao et al. to classify superlattices with different morphologies.^20^ In this system, the *a*-MoO_3_ layer enables strain relaxation due to its two-dimensional (2D) van der Waals flexibility and also passivates dangling bonds at the interfaces. As a result, sharp interfaces were achieved and high electron mobility of up to 71 cm^2^ V^–1^ s^–1^ was reported. However, this demonstration does not fully capture the broader scope of materials in Tsu’s original proposal, particularly for chalcogenide-based material systems. These observations indicate that heteromorphic superlattice structures could be further explored and realized using alternative deposition techniques.

Atomic layer deposition (ALD) has exceptional advantages, including unparalleled control over thickness at the atomic scale, excellent conformality over high-aspect-ratio structures, and low-temperature processing capability. However, the potential of ALD for quantum superlattice engineering remains largely untapped, with most efforts to date focused on oxides.^21,22^ Paul et al. reported bandgap modulation via quantum confinement effects in ALD-grown Al_2_O_3_/TiO_2_ superlattices by precisely controlling the thickness and composition. They reported a significant blueshift of the absorption edge, resulting in an increase in the bandgap from 3.4 to 4.1 eV.^23^ This example emphasizes the potential of ALD for designing tunable optical materials because of its ability to control the layer thickness well below the excitonic Bohr radius, rendering it suitable for achieving strong confinement. In practice, however, experimental studies of nonoxide semiconductor-based superlattices for quantum confinement by ALD have not been reported, revealing an important gap in the field. The key challenges lie in maintaining well-defined interfaces and crystallinity in ALD-grown semiconductor multilayers. Although ALD provides atomic-level thickness control, interfacial roughness and interdiffusion often hinder the formation of well-defined heterostructures or superlattices.^24,25^ In addition, many ALD-grown films lack sufficient crystallinity, which limits the observation of distinct quantum confinement effects.

Among chalcogenide materials, lead sulfide (PbS) is a prototypical material for studying quantum confinement because it has a large Bohr radius of 18 nm and a bandgap energy of 0.4 eV.^26–29^ Accordingly, numerous studies have investigated bandgap modulation in PbS nanocrystals or superlattices to improve application performance.^30–34^ Kowalczyk et al.^200^ demonstrated MBE-grown PbS/EuS superlattices exhibiting quantum size effects accompanied by strain-induced bandgap shifts. However, epitaxial growth techniques such as MBE typically require lattice-matched single-crystal substrates (e.g., BaF_2_ or NaCl). Other PbS-based superlattice approaches have largely focused on nanocrystal assemblies, which often involve organic ligands and are less suitable for scalable thin-film device architectures.^35–37^ In contrast, ALD enables the formation of multilayer structures over large areas on a wide variety of substrates under CMOS-compatible conditions.

For PbS-based heteromorphic combination, tin disulfide (SnS_2_) is an excellent barrier material for electron confinement, due to its wide bandgap, chemical stability, and favorable band alignment. Brontvein et al. synthesized PbS/SnS_2_ nanoparticles using a solar furnace with a temperature of approximately 3000 K and analyzed the superstructure characteristics of misfit layer compounds.^30^ Moreover, Bai et al. reported that the formation of a superlattice with PbS and SnS_2_ significantly altered the phonon dispersion, resulting in hybridization between the acoustic mode and the optical mode.^38^ These findings suggest that the PbS/SnS_2_ superlattice provides a versatile platform for engineering quantum confinement and band structure modulation through periodic potential and interfacial effects.

In this study, we demonstrate the first chalcogenide-based heteromorphic superlattice, which also represents the very first heteromorphic superlattice grown via an industry-compatible ALD method. It consists of *pc*-PbS/*a*-SnS_2_ layers, and experimentally reveal clear quantum confinement effects. Previous studies have reported ALD processes for PbS and SnS_2_ thin films, which exhibited controlled growth and high film quality.^27,39,40^ Considering the structural advantages in previous heteromorphic systems, these findings suggest that ALD can also produce well-defined superlattice thin films with comparable characteristics. The *a*-SnS_2_ layers in the heteromorphic structure effectively passivate dangling bonds at the interface and grain boundaries, preventing interdiffusion and relieving strain stacking in *pc*-PbS. It enables the realization of quantum confinement through a multiple quantum well (MQW) architecture. This work not only bridges a critical gap in ALD but also establishes a scalable framework for designing next-generation quantum and electronic materials. It also provides a practical pathway toward realizing the heteromorphic superlattice concept originally envisioned by Tsu beyond the limitations of conventional epitaxy.

## 2 Results and Discussion

The growth behavior of individual PbS and SnS_2_ thin films were investigated as a function of deposition temperature (Figure S1). The growth per cycle (GPC) remained saturated over the examined temperature range, confirming self-limited surface reactions. Among the investigated temperature, 85 °C was selected because it produces the smoothest PbS films, which supports to form a fine interface, as shown in Figure S2. In addition, maintaining a relatively low process temperature is advantageous for suppressing thermodynamically driven interdiffusion at the interfaces. The PbS/SnS_2_ superlattice was fabricated by alternately depositing PbS and SnS_2_ layers using the ALD supercycle process, which were repeated for four supercycles. To complete the structure, a PbS layer with the same number of sublayer ALD cycles was deposited as a capping layer. The detailed superlattice parameters are summarized in Table S1. The surface morphology of PbS thin films grown at different temperatures is shown in Figure S2.

A heteromorphic structure is a combination of materials with different morphologies. This combination can result in unique interfacial properties and structural stability in superlattices. The heteromorphic superlattice structure, composed of periodically alternating *pc*-PbS and *a*-SnS_2_ layers deposited by thermal ALD, is shown in Figure 1a. The superlattice thin films investigated in this study are stacked beginning with crystalline as-grown PbS, in which Pb and S atoms occupy cubic lattice positions, which is consistent with previous reports on ALD-grown PbS.^19,27,39^ In the *a*-SnS_2_ layer, Sn and S atoms are located without long-range crystallinity. As shown in Figure 1b, the ALD-grown PbS exhibited a cubic crystal structure (JCPDS No. 5–592). With respect to the X-ray diffraction (XRD) patterns of the superlattice, only diffraction peaks corresponding to PbS were observed, and no secondary crystalline phases were observed. This finding suggests that no undesired phases formed at the interfaces. Notably, no peak shifts were observed compared with those of the PbS and PbS/SnS_2_ superlattices (also shown in Figure S3 for superlattices with different periodicities), indicating a lack of strain. Typically, when two materials with different lattice structures are combined, strain can significantly affect the superlattice properties.^5,41^ However, the *a*-SnS_2_ layers act to suppress strain accumulation in the *pc*-PbS layers, reducing lattice distortion.^19^

**Figure 1:**
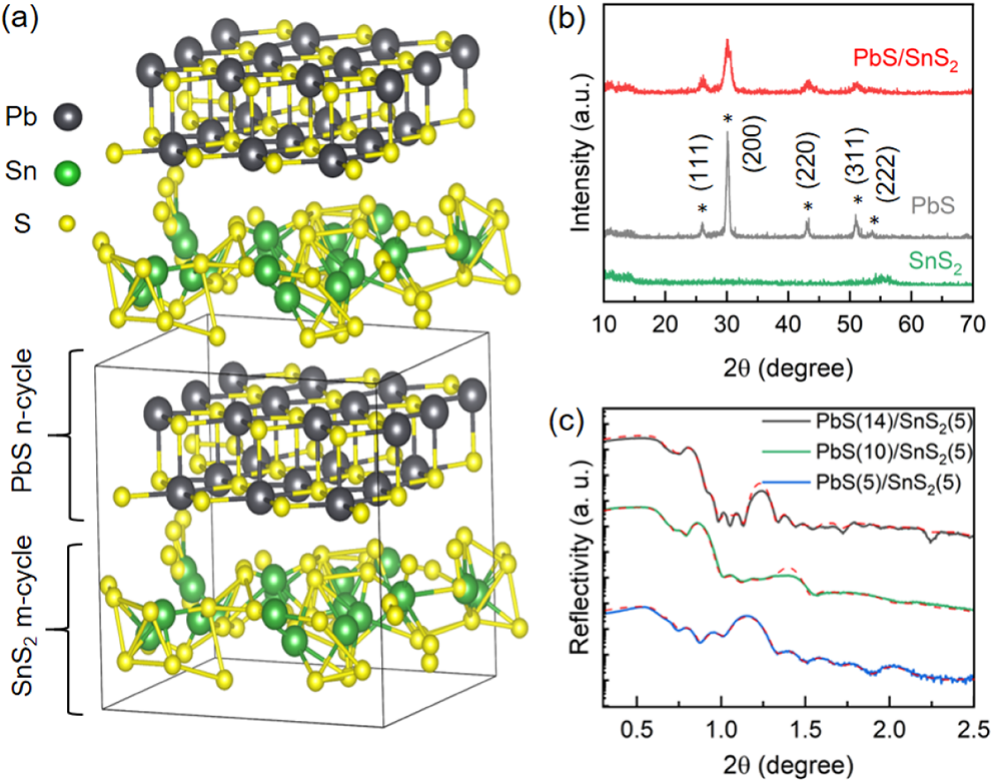
Structural characterization of heteromorphic *pc*-PbS/a-SnS_2_ superlattice thin films. (a) Schematic of a heteromorphic superlattice with two supercycles consisting of *n* and *m* ALD cycles for each PbS and SnS_2_ sublayer. (b) XRD patterns of individual PbS, SnS_2_ and PbS/SnS_2_ superlattices, revealing the lack of SnS_2_ diffraction peaks and the presence of distinct PbS patterns from the PbS/SnS_2_ superlattice. (c) XRR spectra of PbS/SnS_2_ superlattices with different sublayer thicknesses at the nanometer scale, revealing distinct superlattice reflections. The dashed lines indicate the fitting results.

The X-ray reflectivity (XRR) curves of PbS/SnS_2_ superlattices with various sublayer thicknesses are shown in Figure 1c. All the superlattice films, including those not shown in the figure, exhibited superlattice Bragg reflections, which originated from the periodic interfaces between the two distinct sublayers. The thicknesses obtained from the fitting of different superlattice films are well matched to the intended values. The measured XRR curve fitting provides structural parameters such as the density, thickness and roughness of the multistructured films. The densities of the PbS and SnS_2_ layers are ∼6.9 g/cm^3^ and ∼3.5 g/cm^3^, respectively. The corresponding interface roughnesses and sublayer thicknesses for the superlattices are summarized in Table 1. The average interface roughness is higher for thicker PbS sublayers due to the presence of polycrystalline grains, while preserving the integrity of the superlattice interfaces.

**Table 1:** XRR Fitting Parameters Including Density, Interface Roughness, and Sublayer Thickness of PbS/SnS_2_ Superlattices with Different Sublayer Thicknesses

	Density [g/cm^3^]			Sublayer thickness [nm]	
Samples	PbS	SnS_2_	Interface roughness [nm]	PbS	SnS_2_
PbS(5)/SnS_2_(5)	6.9	3.5	0.7	4.4	4.5
PbS(10)/SnS_2_(5)			1.9	9.4	4.4
PbS(14)/SnS_2_(5)			1.8	13.0	4.0

To examine the nanostructure of ALD-grown heteromorphic PbS/SnS_2_ superlattices, cross-sectional transmission electron microscopy (TEM) investigations were performed. As shown in Figure 2a, a clearly resolved layered structure of the uniformly deposited PbS (3 nm)/SnS_2_ (5 nm) superlattice thin film is observed across the entire examined area in the bright-field TEM cross-sectional image. The high-resolution TEM image in Figure 2b reveals a well-defined superlattice structure consisting of alternating PbS and SnS_2_ layers. The superlattice thin films were fabricated via repeated supercycles, each starting with PbS, followed by SnS_2_, and capped with a final PbS layer. A magnified view of PbS is 3 nm in size and oriented along the [100] zone axis within the polycrystalline structure. When thin films grow as-crystalline during the ALD process, island growth often results in a rough surface, which impedes the formation of a smooth and even surface for subsequent layer deposition.^42^ This hinders the development of well-defined superlattices. However, in this work, the amorphous SnS_2_ layer uniformly covered the PbS surface after deposition, promoting the growth of a nicely ordered superlattice (as shown in Figure S4). The depth profile through the film is shown in Figure 2c, indicating that the measured layer thicknesses are consistent with both the intended nominal values and the XRR curves. This finding also suggests that no significant interdiffusion or interfacial exchange reactions occurred during the supercycle process in ALD. The negligible intermixing can be attributed to the low-temperature deposition that suppressed the diffusion of Sn and S into the strong bond of the Pb–S matrix.^43^ The polycrystalline cubic structure of the PbS layers is shown in Figure 2d. The Fourier transform reveals ring radii that correspond to the (100), (111), and (200) lattice plane distances.

**Figure 2:**
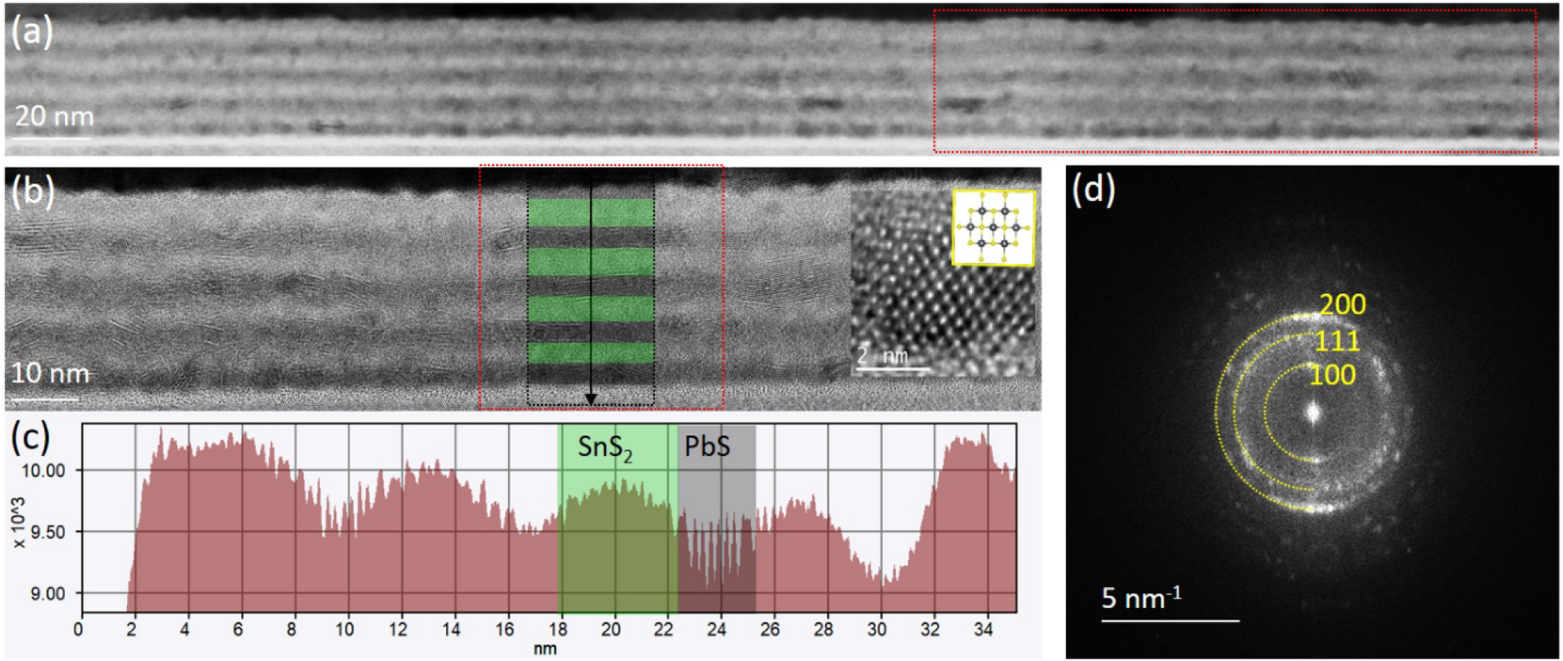
TEM analysis of heteromorphic PbS/SnS_2_ superlattice thin film cross sections. (a) Bright-field TEM image of a representative PbS (3 nm)/SnS_2_ (5 nm) superlattice thin film region, exhibiting uniform deposition over a large area. (b) High-resolution TEM image superimposed with a schematic of the sublayer stacking in gray (PbS)–green (SnS_2_). The inset shows a magnified view of the PbS sublayer and an atomic model of the cubic PbS unit cell. (c) Depth profile along the line scan represented in (b) by the black arrow, revealing distinct thicknesses for each sublayer. (d) Fourier transform (FT) obtained by averaging several FTs of quadratic subimages of (b) with indexed rings corresponding to the (100), (111), and (200) crystallographic planes.

Raman spectroscopy was performed to elucidate the modification of the lattice dynamics and phonon selection rules by the artificial periodicity and heteromorphic interfaces in PbS/SnS_2_ superlattices. The Raman spectra of PbS/SnS_2_ superlattices with different PbS thicknesses in the superlattice period are shown in Figure 3a. The prominent mode at ∼305 cm^–1^ corresponds to the A_1g_ optical phonon mode of SnS_2_,^40^ which originates from the out-of-plane vibrational mode, confirming that the local bonding environment of SnS_2_ remains intact across all the superlattice periods. Additional Raman features at ∼69, 94, and 195 cm^–1^ are attributed to the PbS transverse optic (TO), mixed longitudinal and transverse acoustic (LA/TA), and longitudinal acoustic (LA) phonon modes, respectively.^44–46^ Corresponding peaks were also observed in the spectra of individually grown PbS in this work (Figure S5). These observations also confirm that both PbS and SnS_2_ phases remain structurally intact within the heteromorphic superlattice architecture, consistent with the XRD results. As the PbS sublayer thickness decreases within one PbS/SnS_2_ superlattice period, a systematic shift is observed for the Raman peak at ∼195 cm^–1^ (LA), which is more distinct than that at ∼69 cm^–1^ (TO). This behavior indicates that phonon modes involving larger out-of-plane atomic displacements are particularly sensitive to interfacial effects. When the PbS thickness is reduced, the vibrational eigenvectors increasingly sample the PbS/SnS_2_ interfaces, where altered bonding environments and modified force constants associated with Pb–S–Sn coordination result in phonon renormalization.^47^ Consequently, the Raman response progressively deviates from that of bulk PbS and reflects the emergence of a coupled multilayer vibrational system.

**Figure 3:**
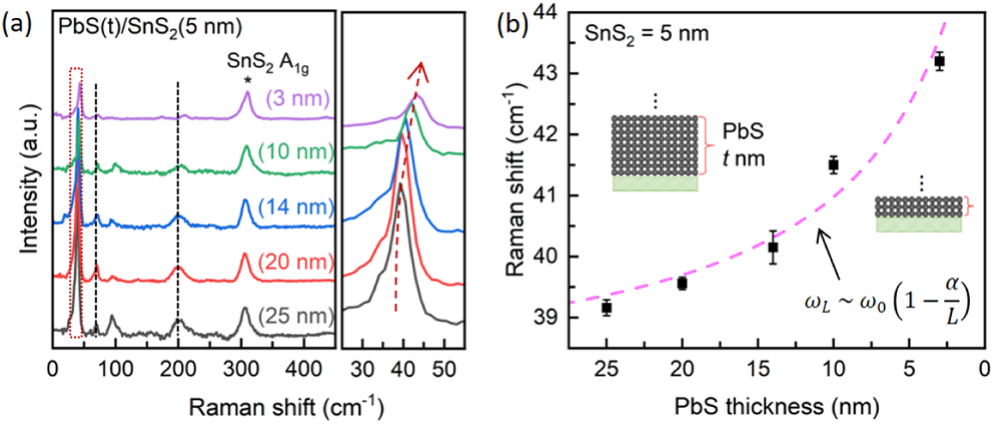
Raman mode evolution in the PbS/SnS_2_ superlattice with different superlattice periods. (a) Raman spectra of superlattices with different PbS sublayer thicknesses within one superlattice period. Intensities were normalized to the SnS_2_ A_1g_ mode. The red dashed box highlights the zone-folding-induced low-frequency Raman mode, shown in a magnified view on the right. The two dashed lines indicate PbS Raman modes: the first dashed line indicates a systematic shift with sublayer thickness, and the second dashed line indicates a nearly constant shift. (b) The shift in the activated low-frequency mode with different superlattice periods. The dashed line represents a fit based on Brillouin zone folding.

Additionally, a striking low-frequency Raman mode centered between ∼39 and 44 cm^–1^ was observed, which was not observed in individually grown PbS and SnS_2_ films. Low-frequency Raman modes are highly sensitive to collective lattice motions and interlayer interactions; thus, their activation implies superlattice-specific phonon characteristics.^48^ Bai et al. reported that the PbS/SnS_2_ superlattice architecture promotes strong hybridization between the acoustic phonons of heavy PbS sublayers and the optical phonons of lighter SnS_2_ sublayers, resulting in newly generated interlayer shear and breathing modes with characteristic frequencies near the Brillouin zone center.^38^ In particular, phonon calculations revealed an interlayer breathing mode at 1.434 THz (∼47.8 cm^–1^). In close agreement with the low-frequency Raman mode observed in this study, these findings confirm its assignment to a superlattice-induced interlayer vibration. The activation and evolution of this low-frequency mode can be elucidated within the framework of zone folding induced by artificial superlattice periodicity. The introduction of a well-defined superlattice period increases the real-space unit cell along the growth direction, reducing the Brillouin zone and folding phonon branches from finite wavevectors back to the Γ point.^49^ As a result, acoustic phonons that are Raman inactive in parent materials can become Raman active in the superlattice when translational symmetry is broken and momentum conservation rules are relaxed. This mechanism is particularly effective for low-frequency acoustic modes, whose dispersion is strongly affected by periodic modulation. As shown in Figure 3b, the frequency of the activated low-frequency mode systematically shifts to higher wavenumbers as the superlattice period decreases. This trend is consistent with zone-folding physics, as confirmed by fitting using the following relation: $\omega_{L}∼\omega_{0}\left(1-\frac{\alpha }{L}\right)$ , where ω represents the wavenumber, *L* denotes the superlattice period, and α indicates a constant.^50,51^ Specifically, decreasing the superlattice period increases the folding wavevector, causing the folded phonon mode to probe steeper regions of the parent acoustic dispersion, which causes phonon hardening. Simultaneously, the increased density of PbS/SnS_2_ interfaces improves the interlayer force constants and phonon hybridization, further contributing to the upward frequency shift. Crucially, unlike epitaxial crystalline superlattices where lattice mismatch often induces coherent strain, this heteromorphic architecture utilizes *a*-SnS_2_ layers as effective strain-relieving buffers. This is corroborated by XRD measurement showing no observable peak shifts (Figure S3) and the stability of other Raman modes, which reveal no detectable strain variation or lattice constant shifts across different periods.^52^ Collectively, the emergence of low-frequency Raman modes, with frequencies systematically dependent on the superlattice periodicity, provides compelling evidence of superlattice-enabled phonon zone folding accompanied by strong interlayer phonon coupling. These results indicate that ALD-grown heteromorphic superlattices can essentially reshape phonon dispersion and activate new collective vibrational modes.

To investigate potential secondary phases and interfacial charge transfer, X-ray photoelectron spectroscopy (XPS) was performed on PbS, SnS_2_, and PbS/SnS_2_ superlattice thin films. In superlattice structures, interfacial charge transfer alters the electronic band states. To identify these changes, the XPS spectra of the superlattice were compared with those of the individual PbS and SnS_2_ films. The PbS/SnS_2_ superlattice exhibited XPS signals characteristic of both individual PbS and SnS_2_ (Figure S6). The Pb 4f signals from the PbS and PbS/SnS_2_ superlattice films are shown in Figure 4a. For individual PbS, the Pb 4f_5/2_ and 4f_7/2_ peaks are located at 142.4 and 137.5 eV, respectively, as reported in previous studies.^28,53^ In the PbS/SnS_2_ superlattice, the binding energies of the Pb 4f peaks decreased to 142.2 and 137.3 eV, respectively. The Sn 3d spectra of individual SnS_2_ and PbS/SnS_2_ superlattice films are shown in Figure 4b. For individual SnS_2_, the Sn 3d_3/2_ and 3d_5/2_ peaks are located at 494.2 and 485.8 eV, respectively, while peaks in the PbS/SnS_2_ superlattice shifted slightly to 493.9 and 485.5 eV, respectively. The S 2p signals from individual PbS, SnS_2_ and PbS/SnS_2_ superlattice films are shown in Figure 4c. The individual PbS and SnS_2_ films exhibit S 2p_1/2_ and 2p_3/2_ peaks at 162.0 and 160.8 eV and at 162.3 and 161.1 eV, respectively. The S 2p binding energies of the PbS/SnS_2_ superlattice also decreased to 161.8 and 160.6 eV for 2p_1/2_ and 2p_3/2_, respectively. Notably, no obvious additional peak or shoulder was observed in the XPS spectrum of the PbS/SnS_2_ superlattice. This observation further confirms the lack of noticeable alloying or interdiffusion between neighboring PbS and SnS_2_ layers, which is consistent with the XRD and Raman results. In addition, compared with the individual films, the PbS/SnS_2_ superlattice shifted the binding energy to lower values. With respect to heterostructures or superlattices, a decrease in the binding energy of one sublayer element is typically accompanied by an increase in that of the adjacent sublayer, which is often attributed to interfacial charge transfer.^54^ Therefore, in this case, both charge transfer and band structure modifications should be considered simultaneously to better elucidate the interfacial behavior.^41^ Further detailed analysis will be provided in the subsequent section.

**Figure 4:**
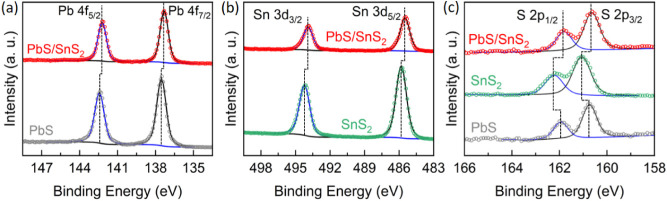
Chemical bonding characteristics via XPS. (a) Pb 4f, (b) Sn 3d, and (c) S 2p states of individual PbS, SnS_2_, and PbS/SnS_2_ superlattices, respectively.

In this study, we present the electronic band structure of the PbS/SnS_2_ superlattice, accounting for the individual PbS and SnS_2_ thin films. The bandgap energies of the individual PbS and SnS_2_ thin films were determined using a Tauc plot from the UV–vis absorption spectra as follows:

1 $${\left(\alpha h\nu \right)}^{n}=A\left(h\nu -E_{g}\right)$$

where α represents the absorption coefficient, *h* denotes Planck’s constant, *A* indicates a constant, ν represents the photon frequency, and *n* = 2 represents a direct transition. As shown in Figure 5a, PbS and SnS_2_ exhibited direct bandgaps of 1.54 and 2.97 eV, respectively.^55,56^ Notably, the bandgap of PbS in this work already falls within a favorable range for efficient optoelectronic applications.^57,58^ This finding is attributed to the intrinsic nanocrystalline effect from deposition, which resulted in lateral confinement.^59^ Moreover, the bandgap value estimated from the Tauc plot closely matches the peak position obtained from the photoluminescence analysis (1.63 eV), further validating the direct bandgap value. The density of states for the PbS cluster as calculated using DFT at the PBE level is shown in Figure 5b, indicating that the observed bandgap originates from lateral confinement. In this work, we separately show the density of states originating from the central and edge regions. The large bandgap, compared to the reported bulk PbS, is a direct result of state confinement caused by the lateral confinement in the cluster geometry, as shown in Figure S7. Furthermore, the edge states located below the lowest unoccupied molecular orbital (LUMO) clearly contribute to the exponential increase observed in the PbS thin film in Figure 5a. The UPS curves of each individual PbS and SnS_2_ thin film are shown in Figure 5c. The cutoff energy in the secondary electron cutoff (SECO) region yields the work function (WF) by the following formula.

2 $$\mathrm{WF}=h\nu -\left|E_{\mathrm{cutoff}}-E_{F}\right|$$

where *h*ν is equal to 21.22 eV, which is the energy of the helium source. The work function values of PbS and SnS_2_ were estimated to be 3.95 and 4.50 eV, respectively. The relative positions of the valence band edge of PbS and SnS_2_ can be estimated from the energy onset in the highest occupied molecular orbital (HOMO) region. On the basis of these values, the electronic band structure of a single quantum well is shown in Figure 5d. Owing to the different work functions, charge transfer occurs from PbS to SnS_2_ when the Fermi energy is aligned. This transfer resulted in a decrease in the XPS binding energy, as shown in Figure 4. However, the simultaneous reduction in binding energy suggests not only interfacial charge transfer but also distinct band bending within the superlattice at the interfaces. Owing to the amorphous nature of SnS_2_, a high density of interfacial defect states acts as traps for electrons transferred from PbS, increasing the built-in electric field and resulting in significant band bending.^60^ This caused the valence band maximum of PbS to approach the Fermi level, resulting in a decrease in binding energy at PbS.^41,61^ Theoretical calculations at the PBE and HSE06 levels further support charge transfer from PbS to SnS_2_, taking into account the band bending at the interface (see Figure S8 and Tables S2 and S3). Nevertheless, the quantum confinement effect was controlled predominantly by the physical thicknesses of the PbS and SnS_2_ layers and the resulting spatial confinement of charge carriers, which will be elaborated in more detail in the following section.

**Figure 5:**
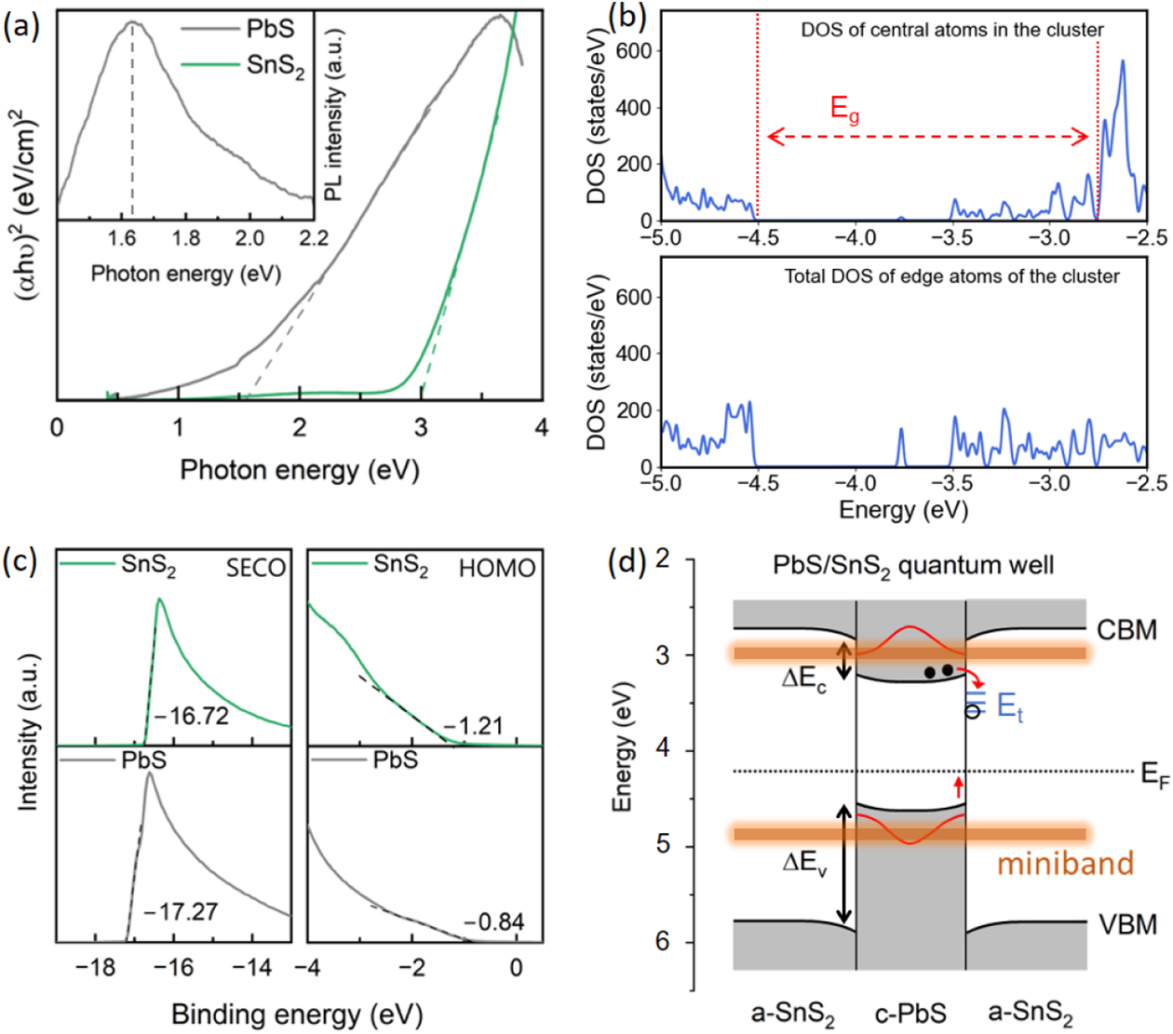
Electronic band structure of PbS and SnS_2_. (a) Tauc plot from the UV–vis measurement of PbS (the inset shows the photoluminescence spectrum). (b) Density of states that are contributed from the different atoms in the central region and edge atoms. The bandgap is shown between the highest occupied molecular orbital (HOMO, left) and the lowest unoccupied molecular orbital (LUMO, right), indicated by red dotted lines. (c) UPS curves for the secondary electron cutoff (SECO, left) and highest occupied molecular orbital (HOMO, right) of PbS and SnS_2_, respectively. (d) Schematic of the electronic band structure of a PbS/SnS_2_ type-I quantum well, illustrating miniband formation and distinct interfacial band bending because of the interface states associated with amorphous SnS_2_.

Quantum confinement generally results in bandgap enlargement because of the quantization of energy states in the well material. This phenomenon becomes especially distinct as the well thickness approaches the exciton Bohr radius, resulting in the formation of discrete energy levels and effective bandgap modulation. The Tauc plot is an effective tool for estimating the bandgap, especially in terms of complex systems such as superlattices.^62^ The bandgap modulation in the superlattice with different PbS thicknesses is shown in Figure 6. Tauc plots were used to extract the bandgap values for the superlattices with different PbS well thicknesses. Notably, the extrapolations were carefully fitted by clearly defined linear regions, which suggests that well-formed minibands arising from quantum confinement effects have been established. A distinct blueshift in the absorption spectra is observed as the PbS thickness decreases, indicating an increase in the bandgap, as shown in Figure 6a. As discussed above, the nanocrystalline effect in PbS was already present because of the lateral confinement effect. PbS with a cubic structure is often grown in a columnar form by ALD (Figure S4), leading to confinement in the lateral direction.^39,63^ Therefore, the increase in the bandgap observed with decreasing PbS thickness was attributed to quantum confinement in the out-of-plane direction, effectively confining electrons along the *c*-axis. In addition, an absorption tail, which is known as the Urbach tail, is observed below the bandgap energy and becomes more distinct with decreasing PbS thickness. The absorption tail originates from nonideal crystalline structures such as edge states, grain size distributions, grain boundaries and defects. When the PbS thickness decreased within one superlattice period, the interface effect became more dominant, resulting in a more distinct absorption tail associated with the interface defect density.^64^ The extracted bandgap values for the superlattice with different PbS thickness are shown in Figure 6b. When the PbS thickness exceeds 18 nm (Bohr radius of PbS), the bandgap remains comparable to that of individual PbS (1.54 eV). However, once the PbS thickness falls below 18 nm, a significant increase in the bandgap, from 1.78 eV (PbS 14 nm) to 2.51 eV (PbS 3 nm), is observed. Additionally, the Tauc plots and bandgap analyses shown in Figure S9 focus on variations in the SnS_2_ barrier thickness to clarify thickness-dependence band modulation. As expected for a quantum well system, the extracted bandgap shows only minor variation with the barrier thickness, indicating that the dominant quantum confinement effect originates from the PbS well layers. The coexistence of strong interlayer coupling and distinct quantum confinement suggests that our heteromorphic system could be categorized within the diffusive Fermi-surface regime.^65^ Furthermore, the bandgap modulation associated with strain is negligible, as shown in Figure S10. The theoretical DFT calculations for PbS, which also confirm the bandgap modulation induced by thickness variation, are shown in Figure 6c and 6d. To elucidate the thickness-dependent bandgap modulation in the out-of-plane direction, we considered a model that is periodic in the lateral plane rather than a finite cluster. The calculated bandgap becomes larger than that of the bulk as the thickness is reduced to the monolayer limit, further confirming the modulation trend controlled by the PbS thickness.

**Figure 6:**
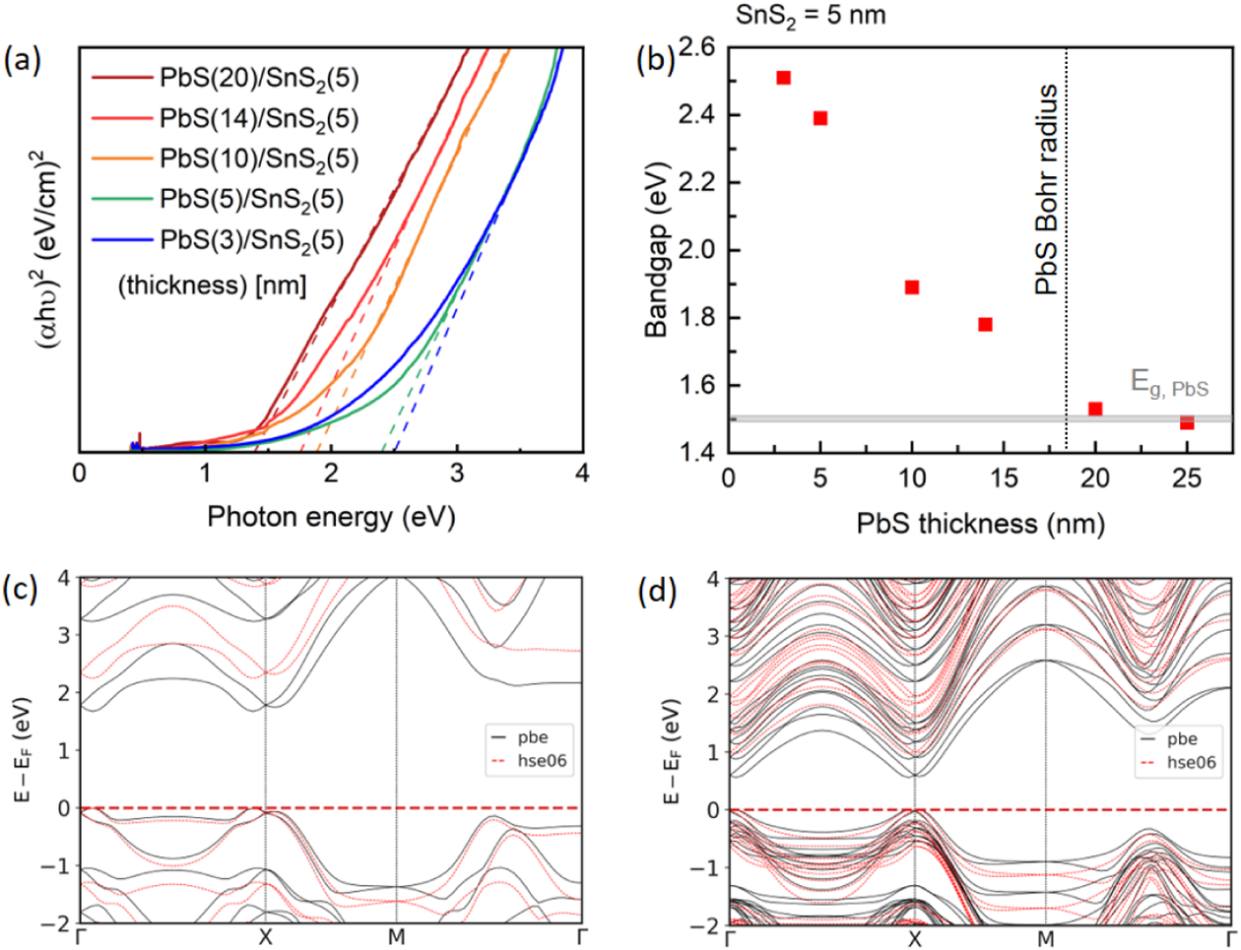
Thickness-dependent bandgap modulation in ALD-grown PbS/SnS_2_ superlattices. (a) Tauc plots extracted from the UV–vis absorption spectra. (b) Optical bandgap energies as a function of PbS well thickness, where the vertical dashed line indicating the Bohr radius of PbS and the onset of quantum confinement. The band structures of PbS for (c) monolayers and (d) pentalayers, simulated by PBE and HSE06. Both calculations confirmed that the bandgap is modulated by the confinement effect.

## 3 Conclusion

In this work, we present the first experimental demonstration of the chalcogenide-based heteromorphic superlattice structure, marking the first utilization of *pc*-PbS/*a*-SnS_2_ via ALD with clear quantum confinement effects. The heteromorphic interface, which is enabled by amorphous passivation, produced sharp and nicely ordered sublayers while effectively relieving strain, as confirmed by XRD, XRR, and HRTEM analyses. Raman investigations further prove the superlattice-induced characteristics via Brillouin zone folding in the low-frequency region and interfacial interactions between PbS and SnS_2_. The chemical bonding states and band structures of individual PbS and SnS_2_ are examined using XPS, UV–vis, PL, UPS, and DFT calculations, comprehensively providing insight into the formation of a type-I quantum well structure. The intrinsic bandgap energies of PbS and SnS_2_ were 1.54 and 2.97 eV, respectively. Theoretical simulations using a PbS cluster model confirmed the presence of lateral confinement effects originating from the polycrystalline nature of ALD-grown PbS. Through modulation of the superlattice periodicity in heteromorphic PbS/SnS_2_ thin films, we successfully engineered the bandgap, which increased from 1.78 to 2.51 eV as the PbS sublayer thickness was reduced below its Bohr radius. The experimental results were consistent with the results of the DFT simulations, highlighting the critical role of thickness-dependent quantum confinement. Overall, this study bridges the knowledge gap between ALD processing and quantum size effects, demonstrating the applicability of heteromorphic ALD superlattices for future electronic and optoelectronic applications.

## 4 Methods

### 4.1 Film Deposition

PbS, SnS_2_ and PbS/SnS_2_ superlattice films were deposited on Si and glass substrates for various characterization techniques. The substrates were cleaned in a sonication bath before deposition using acetone, isopropanol, and deionized water for 5 min each, after which they were dried with a nitrogen gun. UV-ozone treatment was performed for 10 min to remove residual organic contaminants. The films were deposited using a homemade thermal and temporal-mode ALD reactor. (*N*,*N*-Bis­(trimethylsilyl)­acetamidinate)­lead­(II) [Pb­(btsa)_2_], tetrakis­(dimethylamino)­tin­(IV) [TDMASn], and H_2_S (3 vol % in argon) were used as Pb, Sn, and S precursors, respectively. Pb­(btsa)_2_ was dissolved in hexamethyldisilazane (HMDS) to achieve a stable liquid phase. Purified nitrogen gas was used as the carrier gas at a flow rate of 5 sccm. The deposition temperature was 85 °C, and the ALD precursor lines were maintained at 83 °C. Pb­(btsa)_2_ and TDMASn were maintained at 80 and 50 °C, respectively. The detailed ALD processes for PbS and SnS_2_ are presented in Table S4. The PbS/SnS_2_ superlattices were deposited starting with a PbS sublayer and followed by SnS_2_, repeated for four supercycles. A PbS layer with the same number of subcycles was deposited as the final capping layer.

### 4.2 Characterizations

The film thicknesses were measured using a profilometer (Dektak XT-A, Bruker). Surface morphologies and cross-sectional images were obtained using scanning electron microscopy (Sigma 300-ZEISS FESEM) and atomic force microscopy (AFM, ICON, Bruker). Film crystallinity was characterized by X-ray diffraction (XRD with Cu Κα radiation; SmartLab SE, Rigaku). Superlattice structural features were examined by transmission electron microscopy (TEM, Titan 80–300 and Talos F200X, FEI) and X-ray reflectometry (XRR, Panalytical X’pert Pro diffractometer). The measured XRR curves were fitted using an alternating layer stack model in X’pert Reflectivity software. High-resolution transmission electron microscopy (HRTEM, Thermo Fisher Scientific) imaging was performed using an aberration-corrected TF Titan^3^80–300 microscope operated at an acceleration voltage of 300 kV. A cross-sectional TEM lamella of the PbS/SnS_2_ superlattice was prepared using focused ion beam milling (FIB; Helios 5 CX; Thermo Fisher Scientific). Raman spectra and photoluminescence spectra were collected under 532 nm laser excitation (WITec Alpha 300 confocal Raman microscope). The chemical composition and binding energies were investigated by X-ray photoelectron spectroscopy (XPS, ESCALAB 250 Xi by Thermo Scientific). Detailed parameters are provided in Supporting Information. The work function and ionization potential were determined by UV photoemission spectroscopy (UPS, ESCALAB 250 Xi by Thermo Scientific). Bandgap values were estimated using UV–vis spectroscopy (U-3900 spectrometer).

### 4.3 Theoretical Calculations

In this study, the electronic and phonon structures of PbS monoto-penta layers as well as the bulk structure are calculated. Furthermore, the clusters of PbS are simulated. The initial monolayer is retrieved from a computational 2D material database.^66^ The atomic positions and periodic cell sizes of all the structures are numerically relaxed using the BFGS algorithm^67^ as implemented in FHI-vibes^68^ to reach forces below 1 meV/Å. The electronic structure of the systems is calculated within the density function theory (DFT) using the generalized-gradient exchange correlation functional, Perdew–Burke–Ernzerhof (PBE)^69^ and range-separated exchange correlation functional Heyd–Scuseria–Ernzerhof (HSE06),^70^ and hybrid exchange correlation functional PBE0 ^71^ for the selected test cases. Spin–orbit coupling effects are also considered in the calculations,^72^ and the dispersion interaction is modeled using a nonlocal many-body dispersion method,^73^ as implemented in FHI aims.^74^ Additionally, the in-plane quantum confinement was checked using calculations of quadratic clusters of PbS with different side lengths (1 to 10 unit cells). We determined that the ALD-grown amorphous SnS_2_ layer has a large bandgap of 2.97 eV on the basis of the temperature of the growth.^75^ Therefore, it can be modeled as an insulating layer (here, vacuum) between semiconducting PbS layers.

## Supplementary Material



## Notes

Additional information, including supplemental deposition characteristics, SEM images, XRD spectra of the superlattices, and the unit cell used for DFT calculations (PDF)
