# Supplementary material for: Quantum Confinement Effect in a Heteromorphic PbS/SnS2 Superlattice Grown by Atomic Layer Deposition
Source: ACS Nano. 2026 Jun 11;20(24):17373–83. doi: 10.1021/acsnano.6c02718 (PMC13296605; doi:10.1021/acsnano.6c02718)
Supplement: Supplementary file 1 [file nn6c02718_si_001.pdf]

Supporting information

Quantum Confinement Effect in a Heteromorphic PbS/SnS_2 Superlattice Grown by Atomic Layer Deposition

Dong-Ho Shin [a,b], Mohammadreza Daqiqshirazi [d,f], Amin Bahrami [a], Sebastian Lehmann [a], Daniel Wolf [a], Axel Lubk [a], Angelika Wrzesińska-Lashkova [a,e], Yana Vaynzof [a,e], Golam Haider [a], Thomas Brumme [d], and Kornelius Nielsch [a,b,c]

[a] *Leibniz Institute for Solid State and Materials Research, 01069 Dresden, Germany*
[b] *Institute of Applied Physics, Technische Universität Dresden, 01062 Dresden, Germany*
[c] *Institute of Material Research, Technische Universität Dresden, 01062 Dresden, Germany*
[d] *Chair for Theoretical Chemistry, Technische Universität Dresden, 01069 Dresden, Germany*
[e] *Chair for Emerging Electronic Technologies, Technische Universität Dresden, 01187 Dresden, Germany*
[f] *Center for Advanced Systems Understanding, CASUS, HZDR, 02826 Görlitz, Germany*

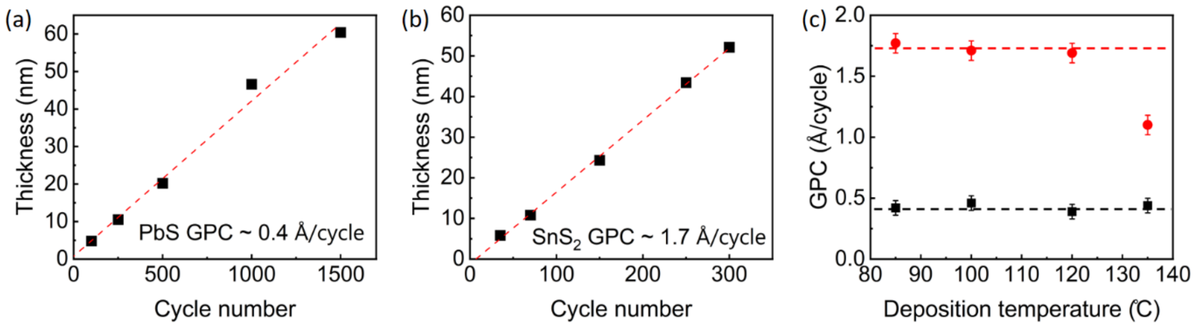

Figure S1. Linear growth with varying ALD cycle numbers for (a) PbS and (b) SnS_2, and (c) saturated growth rate at different deposition temperatures. Both ALD processes for PbS and SnS_2 shows desirable deposition window under the reported temperatures.

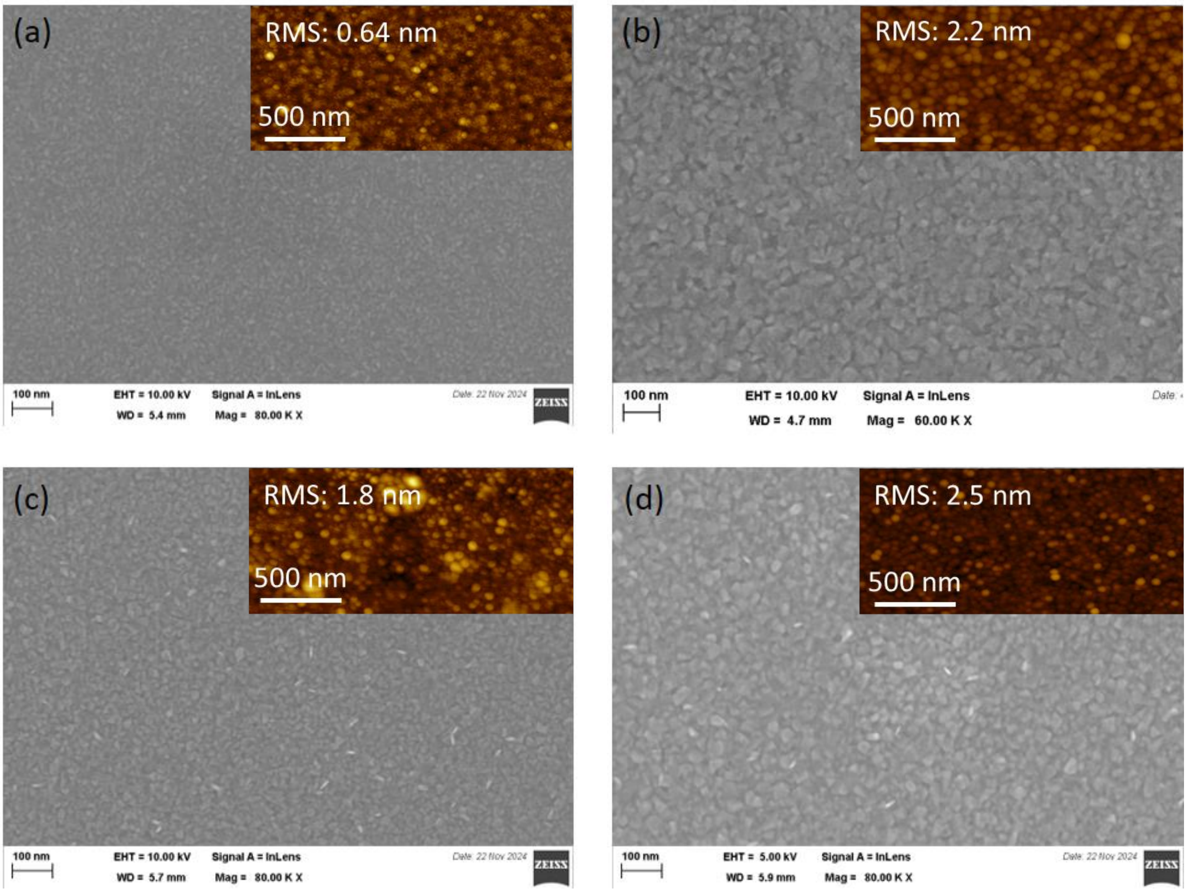

Figure S2. SEM and AFM images showing the surface morphology of PbS thin films deposited at different temperatures: (a) 85 °C, (b) 100 °C, (c) 120 °C, and (d) 135 °C. Among these, 85 °C was selected as the deposition temperature due to its smoother film surface compared to the higher-temperature conditions.

Table S1. Detailed ALD processes for PbS/SnS_2 superlattices with varying thicknesses. The supercycle was repeated four times, ending with PbS cycles as the top layer.

Samples	Cycle numbers		Thickness [nm]
	PbS	SnS_2	
PbS	1500	-	60
$PbS(3)/SnS_2(5)$	75	35	35
$PbS(5)/SnS_2(5)$	125	35	44
$PbS(10)/SnS_2(5)$	250	35	65
$PbS(14)/SnS_2(5)$	350	35	103
$PbS(20)/SnS_2(5)$	500	35	115
$PbS(25)/SnS_2(5)$	625	35	137
$PbS(10)/SnS_2(11)$	250	70	92
$PbS(10)/SnS_2(3)$	250	20	52

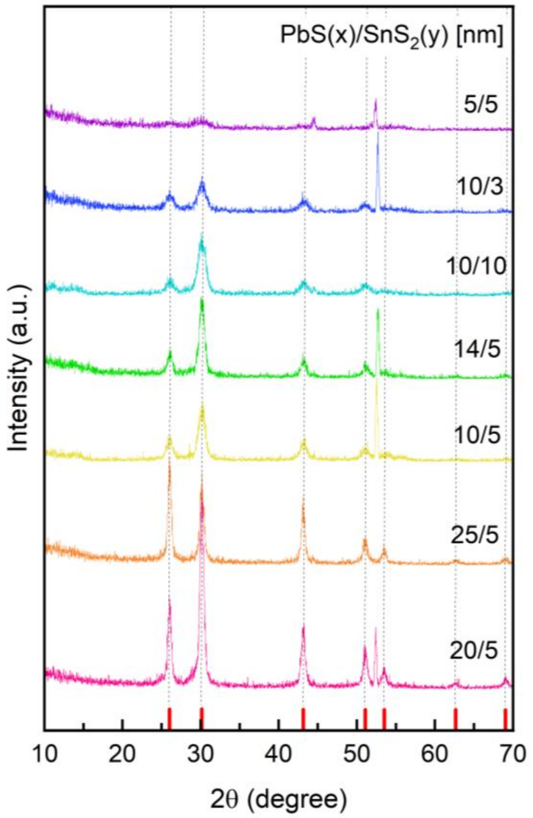

Figure S3. XRD patterns of PbS/SnS_2 superlattices with varying periods, showing only the crystalline peaks of PbS. The absence of peak shifts indicates that strain effects are negligible. (Red ticks: reference PbS pattern from ICSD 5533)

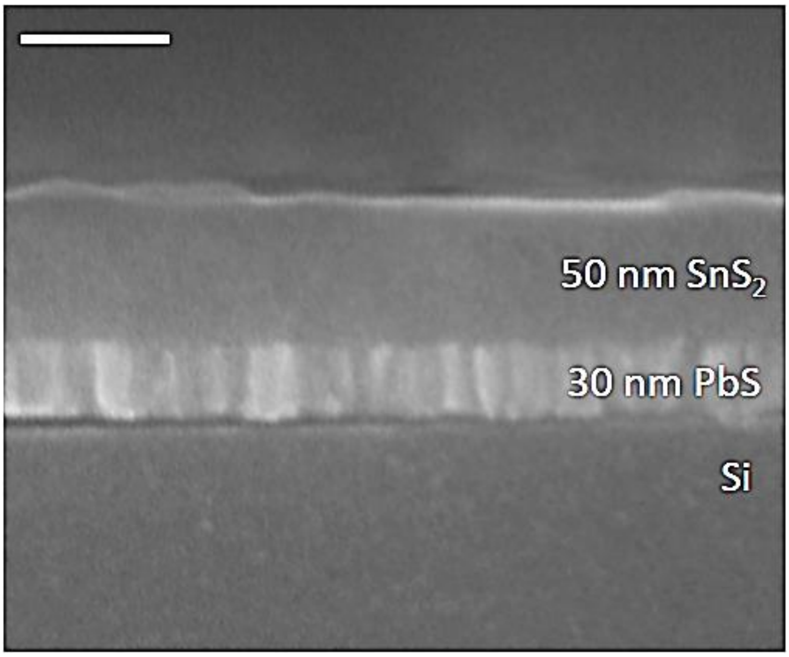

Figure S4. SEM cross-section of ALD-grown PbS/SnS_2 heterostructure, exhibiting a fine interface between a columnarly grown PbS and amorphous SnS_2. (scale bar 50 nm)

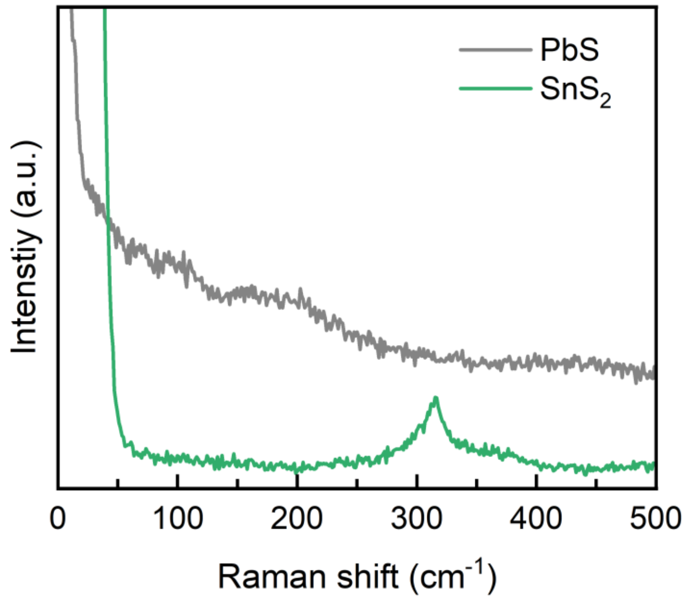

Figure S5. Raman shift of individually ALD-grown PbS and SnS_2.

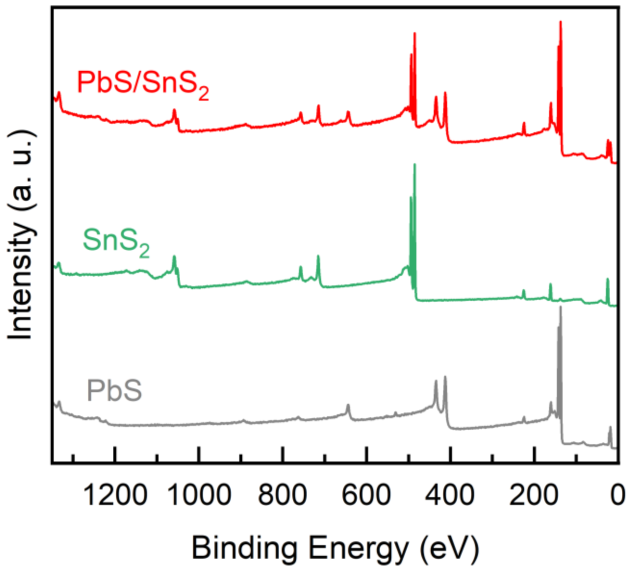

Figure S6. XPS surveys of PbS, SnS_2 and PbS/SnS_2 superlattice.

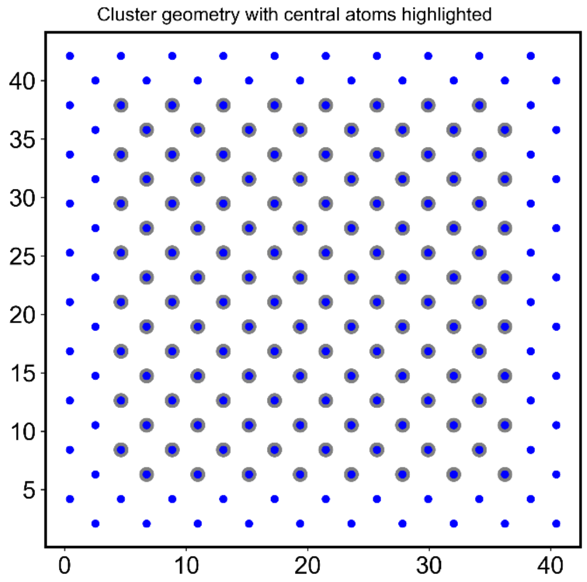

Figure S7. Atom positions of PbS cluster of size 10*10 unitcell with the central atoms being highlighted.

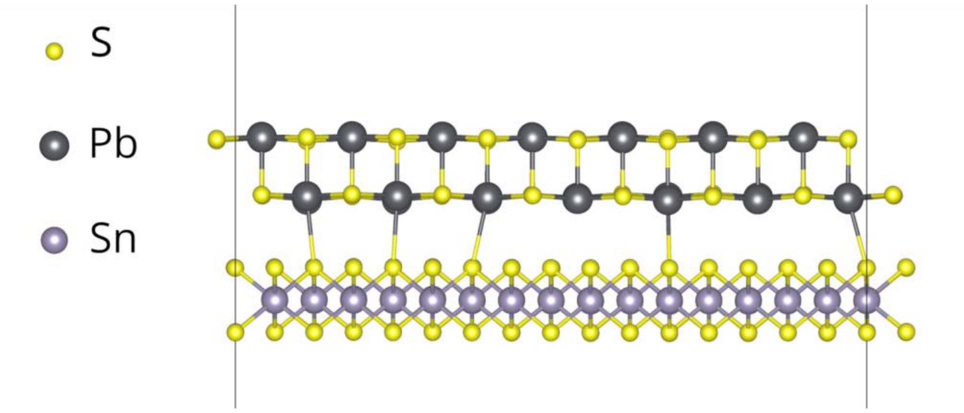

Figure S8. Side view of the PbS/SnS_2 heterostructure.

Table S2. Partial Hirshfeld charges averaged of the PbS/SnS_2 heterostructure for each element baesd on their layers.

Layers	atoms	PBE (avg)	HSE06 (avg)
SnS_2	S	-0.146	-0.162
	Sn	0.287	0.324
	S	-0.143	-0.150
PbS	Pb	0.287	0.307
	S	-0.283	-0.321
	S	-0.299	-0.332
	Pb	0.298	-0.332

Table S3. Partial Hirshfeld charges of the PbS/SnS_2 heterostructure under vertical homogeneous electric field.

Layers	atoms	With electric field -0.1 V/A	w/o field
SnS_2	S	-0.152	-0.146
	Sn	0.287	0.287
	S	-0.143	-0.143
PbS	Pb	0.287	0.287
	S	-0.284	-0.283
	S	-0.296	-0.299
	Pb	0.306	-0.298

We calculated the Hirshfeld charges for both monolayers. For the PbS monolayer we find 0.3 and -0.3 for Pb and S atoms, respectively. For the SnS_2 monolayer, the Hirshfeld charges are 0.2934 and -0.1467 for Sn and S atoms, respectively.

We created a heterostructure of one PbS layer and one SnS_2 layer (Figure S8) using hetbuilder[1], ensuring strain to be smaller than 2% in order to model the charge transfer in the system. We relaxed the structure and calculated the Hirshfeld charges using PBE and HSE06. Table S2 summaries the values of the Hirshfeld charge by the type and the position of the atoms. We find that the total charge on the SnS_2 layer in the heterostructure is about -0.002 – while small, this indicates that charge is transferred from PbS to SnS_2 layer. For completeness also values of the Hirshfeld charge using HSE06 functional are given.

As the SnS_2 is amorphous, we expect the charges accumulate on their interface which causes an electric field perpendicular to the heterostructure (see also discussion in the main text). Table S3 shows values of Hirshfeld charges under a homogeneous perpendicular electrical field of -0.1 V/Å. The total Hirshfeld charge on the SnS_2 layer increases to -0.008. This shows the charge accumulation can further facilitate the charge transfer between the layers.

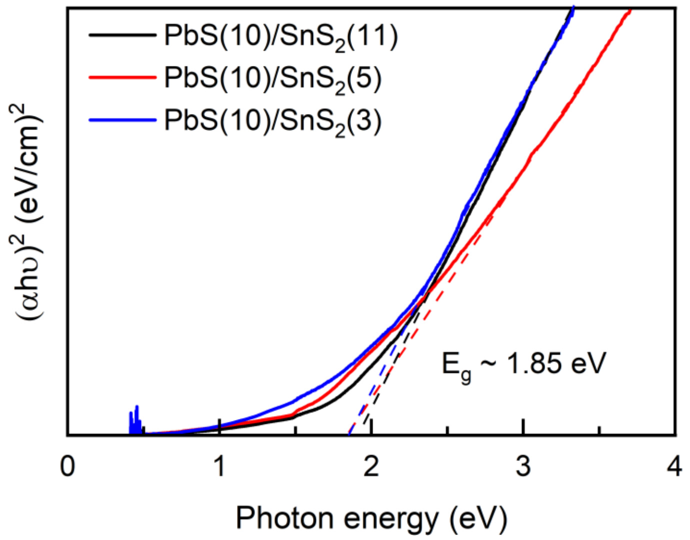

Figure S9. Tauc plots of PbS/SnS_2, obtained from absorption spectra, illustrate the influence of different SnS_2 barrier thicknesses in the superlattice period. The results indicate that varying the SnS_2 thickness does not substantially affect bandgap engineering.

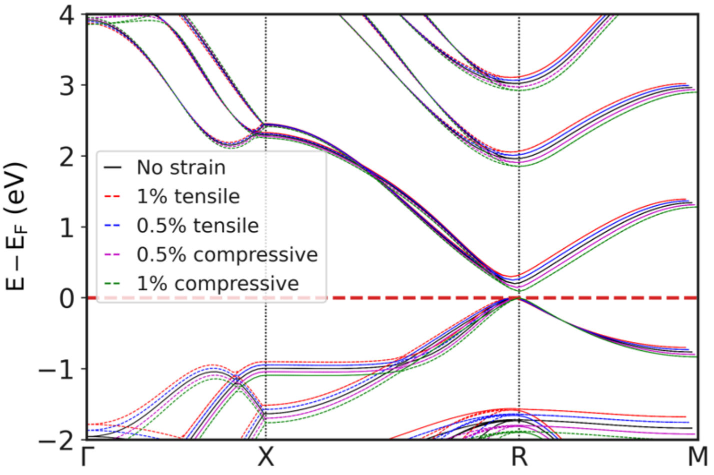

	**Smallest direct gap (eV)**
**-1%**	0.483
**-0.5%**	0.499
**No strain**	0.519
**0.5%**	0.541
**1%**	0.565

Figure S10. DFT-calculated band structures of bulk PbS under varying strain, demonstrating that strain induces only minor changes and thus contributes negligibly to bandgap modulation.

Table S4. The ALD process details of PbS and SnS_2 thin films.

Materials	Precursors (Heating Temp.)	Pulse (s)	Exposure (s)	Purge (s)
PbS	$Pb(btsa)_2$ (80 °C)	2 x 3 times	15	20
	H_2S	0.5		
SnS_2	TDMASn (50 °C)	1	15	20
	H_2S	0.5		

Detailed XPS measurement parameters

X-ray source: XR6 monochromated Al Kα source (hν = 1486.6 eV)

Photoelectron take-off angle: 90° photoelectron take-off angle

Analysis area: 650 µm diameter

Pass energy: 20 eV (high-resolution spectra)

Detector resolution: 0.5 eV full width at half maximum (FWHM) for survey scans

Charge neutralizer: During these measurments, we did not used any charge neutralizer (flood gun), but all spectra were calibrated to the C-C peak at 284.8 eV

Charge referencing: all spectra were calibrated to the C-C peak at 284.8 eV

Data fitting: We used the Smart background, which is an iterative Shirley background with an added constraint to prevent the background from exceeding the measured data at any point. This approach helps to account for both elastic and inelastic electron scattering while ensuring a physically meaningful fit, particularly in complex spectra. The component peaks were fitted using a defined peak model that includes mathematical line shapes and fitting parameters such as peak position, full width at half maximum (FWHM), peak area, Lorentzian character, and, Gaussian character and asymmetry. The final spectrum model was constructed as the sum of all component peaks combined with the selected background.

Reference

[1] Kempt, R. Romankempt/Hetbuilder: Zenodo Release. *Zenodo* **2021**.
